# Personal Responsibility Versus Responsible Options: Health Care, Community Health Promotion, and the Battle Against Chronic Disease

**Published:** 2007-06-15

**Authors:** Joseph R Betancourt, Joan Quinlan

**Affiliations:** The Disparities Solutions Center at Massachusetts General Hospital. (Dr. Betancourt is also affiliated with Harvard Medical School.); Community Benefits at Massachusetts General Hospital, Boston, Mass

During the past century, the health and life expectancy of U.S. residents have improved substantially, largely because of initiatives in public health, including health promotion and disease prevention efforts. Data now suggest that the United States has undergone an epidemiologic transition, in which the leading causes of death are no longer related to infectious diseases but instead to chronic conditions such as heart disease and diabetes. Although much of the progress in reducing the burden of infectious diseases in the United States can be attributed to environmental principles such as the provision of clean water and sanitation and the establishment of food safety standards, many are seeking to abandon these principles as the United States tackles the new epidemic of chronic disease.

In its concern with developing and disseminating new diagnostic and therapeutic modalities — including more effective medications — the U.S. health care system often seems to focus more on treating the disease rather than the patient. The paradigm of personal responsibility for one's health, which includes the responsibility of patients to follow their physician's instructions and adhere to their treatment plan, now carries great weight among health care providers. We've often heard our colleagues say something like, "If we can just get our patients to do what we want them to do, they would be better off." But to them we say this: as you ask your patients to take personal responsibility for their health care, do the society and the health care system of which you are a part provide your patients with appropriate options? For example, can diabetes patients in fact get the healthy foods we instruct them to eat? Are such foods available and affordable in their community? Can heart disease patients exercise safely in their community? Do they even have a sidewalk where they live? Even if asthma patients take their medications, can they rest assured that the mold and dust in their apartment, or the incinerator one block down, or the diesel bus that passes on their street 30 times a day will not make them acutely short of breath?

There is no doubt that social factors often addressed by public health practitioners — such as people's level of education or socioeconomic status, the condition of their housing, the healthfulness of their physical environment, and how they are affected by stress and racism — contribute to health outcomes (1-7). The effect of these "social determinants of health," however, should be the concern of the entire health care community, not just public health practitioners. Anyone who provides health care to people with diabetes, asthma, or heart disease, for example, rapidly realizes the link between their patients' social context and their patients' ability to control their chronic condition. The negative effect of certain social factors on people's health is especially pronounced among some minority groups, and the health disparities that these groups have experienced are now garnering greater attention (8). For example, researchers have shown that three of the five largest landfills in the United States are located in African American or Latino communities and that rates of pediatric asthma near these landfills are among the highest in the country (9). Addressing such racial and ethnic health disparities needs to be a key part of the U.S. health care agenda.

We applaud the Centers for Disease Control and Prevention and the National Expert Panel on Community Health Promotion for their leadership in charting a new path toward community health promotion as described by Navarro et al (10) in this issue of *Preventing Chronic Disease*. Their recommendations are right on target and are a clarion call for all health care professionals: being more attentive to patients' mental health, access to care, and cultural beliefs and values, as well as to the need to develop effective resources for community-level health care, will undeniably improve our ability to address chronic disease in all its complexity. Their article also points out that the success of efforts to improve community health promotion will require a better understanding of community resources, power relationships, political dynamics, long-standing social stressors, and the ability of government agencies and nongovernmental organizations to respond to acute situations such as the 9/11 attack and Hurricane Katrina. It also argues convincingly that conducting research and surveillance, building capacity for community health promotion, investing in new approaches to health promotion, and making appropriate changes in federal health care investments will be key to reducing the impact of chronic diseases.

One important challenge that Navarro et al highlight is how to "emphasize the important role of long-term community health promotion in addressing the social and environmental determinants of health in an atmosphere that demands evidence of health impact and return on investment" (10). The new pay-for-performance contracts provide a financial incentive for hospitals and physicians to engage in such health-promotion efforts. For example, to improve diabetes management for its patients, Massachusetts General Hospital (MGH) has pay-for-performance contracts with health plans that include financial incentives for achieving certain benchmarks in clinical and process measures such as appropriate glucose and cholesterol control among diabetes patients. In addition to using standard quality improvement strategies to achieve these benchmarks (e.g., by providing diabetes decision support to physicians via electronic medical records), MGH has also invested in bilingual case managers, group education visits for patients, and "culturally competent disease management," which focuses on nutrition, exercise, and overcoming sociocultural and environmental barriers that may prevent certain patient populations from optimizing their diabetes control. In short, MGH determined that to improve the care of people with diabetes, it needed to focus not only on what happens in the doctor's office but also on the environmental and social context of its patients. This is the essence of how the concept of return on investment can be incorporated into community health promotion efforts to address chronic disease.

Managers of health care systems need to realize that the complex chronic conditions affecting their patients' health cannot be addressed successfully in the doctor's office alone and that responsible health care isn't just about the personal responsibility of patients but also requires that the health care system itself be responsible in providing health care consumers with appropriate options. These options, however, can be provided only if we partner with local governments, community organizations, and other health and human service providers to develop strategies to address the social, cultural, economic, and environmental determinants of health.
